# Unterstützung der Opioidrotation mithilfe von Online-Apps: Evaluation der Anwendbarkeit und Vergleich mit der LONTS-Leitlinie

**DOI:** 10.1007/s00482-022-00683-5

**Published:** 2022-12-12

**Authors:** Jan D. Wandrey, Niklas Behnel, Sascha Tafelski

**Affiliations:** Klinik für Anästhesiologie mit Schwerpunkt operative Intensivmedizin, Campus Charité Mitte und Campus Virchow-Klinikum, Charité – Universitätsmedizin Berlin, Gliedkörperschaft der Freien Universität Berlin und Humboldt Universität zu Berlin, Charitéplatz 1, 10117 Berlin, Deutschland

**Keywords:** Opioidumrechnung, Medizin-Apps, Opioidtherapie, Opioidanalgetika, Morphinäquivalent, Opioid conversion, Medical applications, Opioid therapy, Analgesics, opioid, Morphine equivalent

## Abstract

**Hintergrund:**

Aufgrund von Arzneimittelnebenwirkungen, Medikamenteninteraktionen oder wegen inadäquater Wirkung bei der Behandlung mit Opioiden kann eine Opioidrotation indiziert sein. Zur Bestimmung der oralen Morphinäquivalenz ist mit der Leitlinie „Langzeitanwendung von Opioiden bei chronischen nicht-tumorbedingten Schmerzen (LONTS)“ ein Praxiswerkzeug veröffentlicht. Dem gegenüber stehen mehrere Apps, die bislang nicht bewertet wurden.

**Material und Methoden:**

Mittels Google Play Store®, iOS App Store® und der Suchmaschine Google® wurden Apps zur Opioidkonversion gesucht. Deutsch- und englischsprachige Apps mit Kalkulatorfunktion wurden eingeschlossen. Mit den Apps wurden 16 Testfälle aus der klinischen Praxis kalkuliert und die Abweichung von der Empfehlung der LONTS-Leitlinie berechnet.

**Ergebnisse:**

Insgesamt wurden 17 Apps identifiziert. Elf benannten die Herkunft des Algorithmus, 3 davon benannten Literaturquellen. Keine App wies ein Qualitätssiegel auf, zudem ließen sich mit keiner App sämtliche Fälle lösen. Es wurden Abweichungen der resultierenden oralen Morphinäquivalente um +179 % von der leitliniengerechten Umrechnung identifiziert. Vier Apps warnten vor Überdosierungen.

**Schlussfolgerung:**

Obwohl die Apps die Umrechnung zwischen Opioiden vereinfachen, besteht eine hohe Varianz der Umrechnungsfaktoren und teils eine große Abweichung von evidenzbasierten Tabellen. Insgesamt besteht ein hohes Risiko von Opioidfehldosierungen.

**Zusatzmaterial online:**

Die Online-Version dieses Beitrags (10.1007/s00482-022-00683-5) enthält eine Tabelle der eingeschlossenen Apps zur Umrechnung von Opioidanalgetika.

## Abstract (en)

**Background:**

Opioid rotation can be indicated due to drug side effects, drug interactions or inadequate effect of treatment with opioids. For the determination of the oral morphine equivalence, a practice tool has been published with the long-term use of opioids in chronic nontumor-related pain (LONTS) guidelines. In contrast, several apps are available that have not yet been evaluated.

**Material and methods:**

Apps and web applications for opioid conversion were searched using Google Play Store®, iOS App Store® and the Google® search engine. German and English language apps with calculator functions were included. Using the apps, 16 test cases from clinical practice were calculated and the deviation from the recommendation of the LONTS guidelines was calculated.

**Results:**

A total of 17 apps were identified, 11 named the origin of the algorithm and 3 of them defined the literature sources. None of the apps and web applications had a quality seal, and none could solve all cases. Deviations of the resulting oral morphine equivalents of up to 179% from the guideline-compliant conversion were identified and 4 apps warned for overdosing.

**Conclusion:**

Although the apps and web applications simplify conversion between opioids, there is high variance in conversion factors and sometimes a relevant deviation from evidence-based tables. Overall, there is a high risk of false opioid dosing.

## Hintergrund und Fragestellung

Opioide sind integraler Bestandteil der Therapie sowohl akuter als auch chronischer Schmerzen [7, 20]. Deshalb sind sie auch in der Liste essenzieller Medikamente der Weltgesundheitsorganisation (WHO) aufgeführt [19]. Insbesondere in der langfristigen Behandlung mit Opioiden kann aufgrund von Nebenwirkungen, Medikamenteninteraktionen, inadäquater Analgesie, Toleranzentwicklung oder opioidinduzierter Hyperalgesie eine Opioidrotation erforderlich werden [7]. Für die Berechnung einer vergleichbaren analgetischen Potenz mit dem neuen Opioid haben sich medikamentenindividuelle Umrechnungs- und Äquivalenzfaktoren etabliert. Dabei wird als Referenzwert die Potenz in oralen Morphinäquivalenten angegeben. Auch wenn in der Praxis häufig Umrechnungstabellen mit festen Faktoren eingesetzt werden, finden sich in der Literatur breite Spannweiten für die Umrechnung der jeweiligen Morphinäquivalente [4, 10, 14, 17, 18]. Eine Empfehlung findet sich im Praxiswerkzeug 10 der Leitlinie „Langzeitanwendung von Opioiden bei chronischen nicht-tumorbedingten Schmerzen (LONTS)“ (https://www.awmf.org/leitlinien/detail/ll/145-003.html), in dem Umrechnungsfaktoren im Konsensusprozess der Mitglieder der Leitliniensteuergruppe veröffentlicht wurden [7].

Apps für die Opioidkonversion sollten laut AkdÄ mit Vorsicht genutzt werden

Dem gegenüber stehen technische Weiterentwicklungen in Form von Smartphoneapplikationen und Webanwendungen (Apps), welche die klinische Arbeit unterstützen sollen. Die Verwendung digitaler Apps im Gesundheitswesen steigt: Laut einer Umfrage gaben 2017 53 % der befragten Ärztinnen und Ärzte an, digitale Apps im Berufsalltag zu nutzen [13]. Es wurden auch Apps speziell für die Opioidkonversion entwickelt, die durch eine direkte Umrechnung von Ursprungs- zu Zielopioid den Rechenprozess vereinfachen und menschliche Fehler bei der Umrechnung minimieren sollen. Eine zehn Jahre alte Studie konnte mit damaligen Smartphone-Apps jedoch eine breite Streuung der Dosierungsempfehlungen feststellen [6]. In einer Stellungnahme zur Arzneiverordnung in der Praxis der Arzneimittelkommission der deutschen Ärzteschaft (AkdÄ) wurde mit Blick auf die Anwendung von Apps betont, dass diese Option mit Vorsicht genutzt werden sollte [14]. Während auf der einen Seite eine Opioidumrechnung mithilfe von Apps Vorteile bieten kann, bestehen auf der anderen Seite potenzielle Gefahren.

Ziel dieser Studie ist es, die Umrechnung von Opioiden durch aktuelle Apps zu evaluieren. Des Weiteren sollen die Umrechnungsergebnisse der Apps mit den Umrechnungen basierend auf Leitlinienempfehlungen verglichen werden.

## Studiendesign und Untersuchungsmethoden

### Suchstrategie für Apps

Die Suche erfolgte für Smartphone-Apps über den iOS App Store® (Apple Inc., Cupertino, CA, USA) sowie den Google Play Store® (Google LLC, Mountain View, CA, USA). Nach Webanwendungen wurde mithilfe der Suchmaschine Google® gesucht. Die Suchbegriffe waren „Opioidrechner“, „opioid calculator“, „Opioidrotation“ und „opioid equivalent estimator“. Die Suche der Apps wurde in 2 Suchsitzungen am 13.08.2021 und am 10.07.2022 durchgeführt. Da der größte Teil der Nutzer:innen lediglich die ersten Ergebnisse betrachtet, wurde die Suche auf die ersten 50 Treffer im iOS App Store® und Google Play Store® sowie die erste Ergebnisseite der Suchmaschine Google® beschränkt [3]. Dabei wurden alle mobilen und webbasierten Apps eingeschlossen, deren Beschreibung eine Umrechnungsmöglichkeit für Opioide angab. In der Folge wurden sämtliche Duplikate entfernt und nur Ergebnisse in englischer oder deutscher Sprache eingeschlossen. Weiterhin wurden in Analogie zur Anwendung in Krankenhäusern Computeranwendungen mit zu installierenden Programmen oder Dateien ausgeschlossen. Passive Umrechnungsangebote wie Tabellen oder Listen wurden ebenfalls ausgeschlossen und nur aktive Kalkulatoren erfasst. Es wurden sowohl kostenpflichtige als auch kostenfreie Apps eingeschlossen.

### Opioidumrechnungen

Zur Testung der Umrechnung der Apps wurden Konversionsbeispiele von Opioidumrechnungen verwendet, die sich in der klinischen Arbeit des Schmerzdiensts der Charité ergeben haben. Die Fälle sind in Tab. 1 aufgeführt.

**Table 1:** 

	Ausgangs‑/Zielopioid mit Darreichungsform	Dosierung des Ausgangsopioids (mg/Tag)	Dosierung des Zielopioids nach Leitlinie (mg/Tag)
1	Oxycodon p.o./Morphin p.o.	80	160
2	Morphin p.o./Oxycodon p.o.	100	50
3	Hydromorphon p.o./Oxycodon p.o.	8	30
4	Oxycodon p.o./Hydromorphon p.o.	60	16
5	Oxycodon p.o./Tapentadol p.o.	20	100
6	Tilidin p.o./Tapentadol p.o.	300	75
7	Tramadol p.o./Morphin p.o.	600	60
8	Tilidin p.o./Oxycodon p.o.	400	20
9	Fentanyl-TTS/Hydromorphon p.o.	75 µg/h	24
10	Oxycodon retard p.o. 160 mg/Tag auf Morphin i.v.	60	n.a.
11	Morphin i.v./Methadon p.o.	28,8	n.a.
12	Methadon p.o./Morphin p.o.	19	n.a.
13	Sufentanil i.v./Hydromorphon p.o	150 µg	n.a.
14	Fentanyl-TTS/Morphin i.v.	25 µg/h	n.a.
15	Hydromorphon i.v./Hydromorphon p.o.	24	n.a.
16	Morphin p.o./Oxycodon p.o.	1200	600

*n.a.* nicht anwendbar, *TTS* transdermales therapeutisches System

Als Standard für die Umrechnung wurde das Praxiswerkzeug 10 der LONTS-Leitlinie genutzt (Tab. 2; [7]).

**Table 2:** 

Opioid	Darreichungsform	Äquivalenzdosis Morphin:Zielopioid
Buprenorphin	Transdermal	1:75
Fentanyl	Transdermal	1:100
Hydromorphon	Oral	1:7,5
Morphin	Oral	Referenz
Oxycodon	Oral	1:2
Tapentadol	Oral	2,5:1
Tilidin	Oral	10:1
Tramadol	Oral	10:1

*LONTS* „Langzeitanwendung von Opioiden bei chronischen nicht-tumorbedingten Schmerzen“

Die Testfälle wurden mit den jeweiligen Apps in die gewählte Zieldosierung umgerechnet. Die Umrechnung wurde auch durchgeführt, wenn eine App nicht zwischen verschiedenen Darreichungsformen differenzierte und somit nur die Opioiddosis konvertierte, ohne verschiedene Bioverfügbarkeiten der Applikationsform zu berücksichtigen. Verlangten Apps die Eingabe von Patientencharakteristika, wurde ein Lebensalter von 50 Jahren verwendet. Bei einigen Apps, die eine Dosisreduktion für eine inkomplette Kreuztoleranz berücksichtigen konnten, wurde zum Zweck der Vergleichbarkeit eine Dosisreduktion von 0 % eingestellt. Weiterhin wurden für die Apps verschiedene Parameter wie das Vorhandensein von Datenschutz- oder Warnhinweisen, die Quellenlage sowie die Qualifikation der Autoren erfasst.

### Statistik

Die Auswertung der deskriptiven Statistik erfolgte mit SPSS (Version 28). Um eine normierte Darstellung der Abweichung der Umrechnung vom Umrechnungsstandard darstellen zu können, wurden die jeweiligen Zielsubstanzen nach LONTS-Leitlinie in orale Morphinäquivalente umgerechnet. Mithilfe von SPSS erfolgte die grafische Darstellung der Abweichungen der Umrechnung der jeweiligen Fälle. Für Testfälle, für die keine Umrechnungsempfehlungen in der LONTS-Leitlinie hinterlegt sind (Fälle 10–15), wurde lediglich die Spannweite der berechneten Ergebnisse dargestellt. Fall 16 wurde nicht statistisch ausgewertet und diente lediglich der Prüfung auf einen eventuellen Warnhinweis der Apps auf eine hohe Opioiddosis. Aufgrund des explorativen Charakters dieser Untersuchung erfolgte keine vergleichende Analyse auf statistische Signifikanz; die Arbeit beschränkt sich auf eine klinische Bewertung der deskriptiven Daten.

## Ergebnisse

### Identifikation von Apps

**Figure 1:**
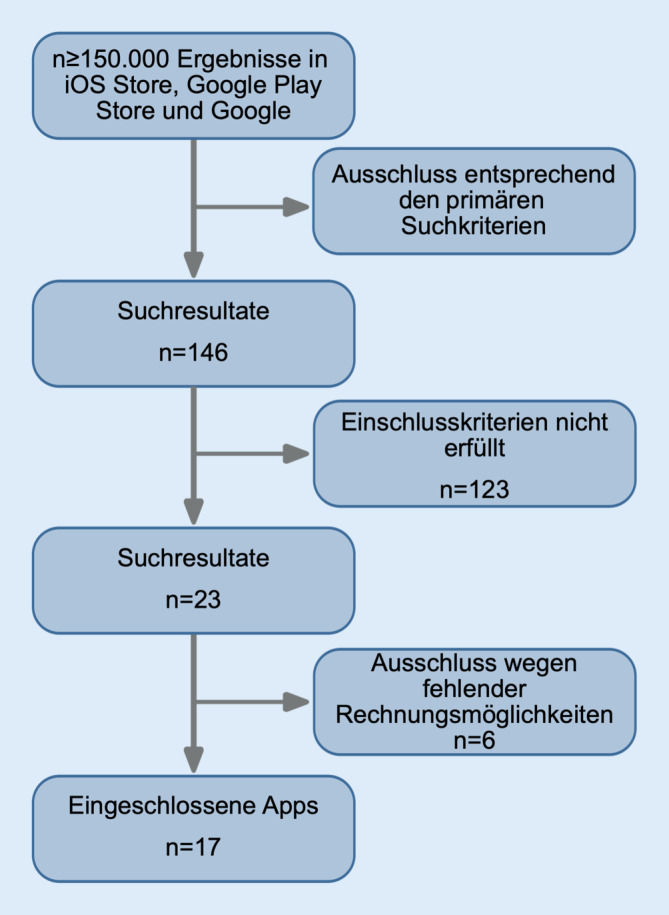

Über die Suchplattformen wurden initial über 150.000 potenzielle Treffer identifiziert. Nach Anwendung der Suchkriterien konnte die Anzahl der Treffer auf 146 reduziert werden. Nach Anwendung der Ausschlusskriterien wurden insgesamt 23 Apps identifiziert, von denen 3 kostenpflichtig waren. Bei einer der Apps war aufgrund einer veralteten iOS-Version der App kein Download mehr möglich. Zwei weitere Apps mussten sekundär ausgeschlossen werden, da sie entgegen der Beschreibung keine Kalkulation ermöglichten. Ebenfalls ausgeschlossen wurden 3 Apps, die lediglich die Umrechnung eines Ausgangsopioids in die orale Morphinäquivalenz ermöglichten. Somit konnten 17 Apps eingeschlossen werden (Abb. 1 und Online-Zusatzmaterial).

Bei der zweiten Suche war eine App (Opioidrechner, Grünenthal GmbH) nicht mehr zum Download verfügbar. Die Ergebnisse der App wurden dennoch in die Studie aufgenommen, da die App in mehreren Publikationen [14, 16] zum Thema Opioidkonversion explizit benannt wurde und damit noch mit einer aktuell gängigen Verwendung gerechnet werden kann.

### Qualitative Charakterisierung der Apps

Keine der identifizierten Apps wies eine Conformité-Européenne(CE)-Kennzeichnung oder die explizite Markierung als Medizinprodukt aus. Vor der Verwendung wurde von einer App die datenschutzrechtliche Zustimmung zur Verarbeitung der Daten konkret verlangt. Zugriff auf die Stammdateien und die Ortungsdienste des Handys war für die Nutzung von 2 Apps notwendig.

Zehn Apps beschrieben, dass eine Ärztin oder ein Arzt in der Entwicklung der App involviert war. In 7 Fällen fehlten klare Angaben zur Autorenschaft. Die Schirmherrschaft einer größeren Organisation oder eines Krankenhauses bei der Entwicklung wurde in 5 Apps angegeben.

Elf Apps benannten Referenzen für die Umrechnung mit großer Varianz. In 6 Fällen konnte keine Umrechnungsgrundlage erkannt werden. Drei der 11 Apps wiesen auf Literaturquellen als Grundlage hin, während 4 Apps Umrechnungstabellen unklarer Herkunft als Quellen benannten. Zwei weitere Apps verwiesen auf nutzerbasierte Internetportale und verlinkten Publikationen sekundär. Eine App verwies auf Wikipedia als Quelle. Eine Publikation bewarb ersatzweise das organisationseigene E‑Learning-Programm zum Thema Opioide. Die zeitliche Aktualität der Quellen wurde in 4 Fällen bezeichnet, die Ursprungszitate wurden dabei mit Quellen aus den Jahren 1966–2018 belegt.

### Quantitative Analyse der Umrechnungen

**Figure 2:**
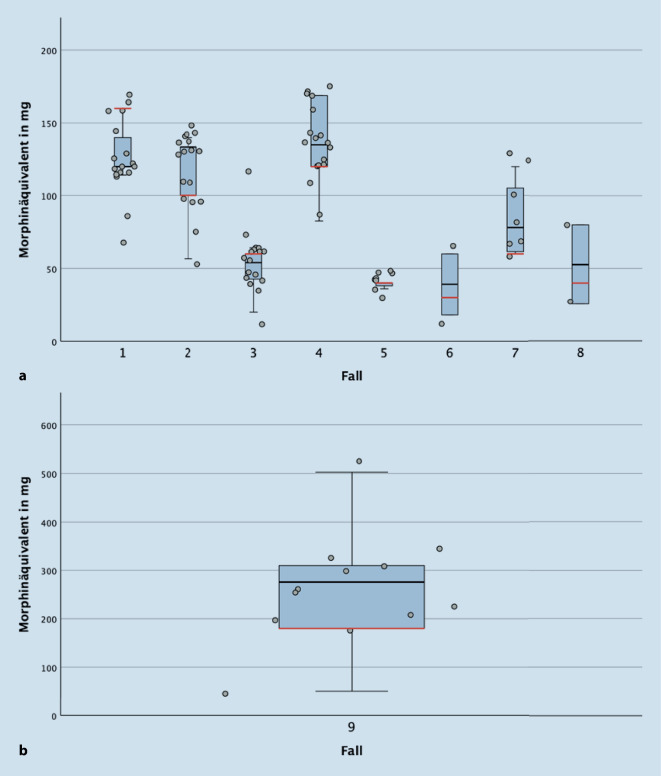

Keine App war zur Berechnung *jedes* Testfalls in der Lage. *N* = 5 der Testfälle konnten von jeder App berechnet werden. Für die Fälle 1–9 war eine Umrechnung nach Leitlinienempfehlung möglich, wobei der Fall 9 im Verhältnis zu den anderen Fällen eine deutlich breitere Streuung zeigte (Abb. 2a,b). Die kleinste prozentuale Abweichung von der Umrechnung nach Leitlinienempfehlung pro Fall betrug im Median 0 % (Fall 5), während die größte im Median bei 53 % lag (95,6 mg orale Morphinäquivalente, Fall 9). Weiterhin konnten Abweichungen um −71 % bis zu +179 % vom Referenzwert nach LONTS-Leitlinie festgestellt werden (−129 mg und +322,5 mg orale Morphinäquivalente, Fall 9).

Für die Fälle 10–16 war keine quantitative Analyse der Abweichung möglich. Eine Umrechnung von Sufentanil in Hydromorphon war in lediglich 4 Apps möglich, wobei im Median als Zieldosierung 20 mg Hydromorphon (Minimum – Maximum 11–60 mg) angegeben wurden. Die Konversion von 28,8 mg Morphin i.v. in Methadon p.o. war in 9 Apps möglich. Dabei wurden im Median 19,1 mg Methadon (Minimum – Maximum 2–23 mg) berechnet. Die Rückkonversion von Methadon in Morphin war in 7 Fällen möglich. Dabei wurden als Zielsubstanz im Median 27,1 mg Morphin (Minimum – Maximum 20–63 mg) von den Apps ausgegeben. Die Umrechnung aus oralem Oxycodon in Morphin i.v. war in 16 der eingeschlossenen Apps möglich. Dabei wurde für die Zielsubstanz eine Dosierung von im Median 80 mg (Minimum – Maximum 65–120 mg) angegeben. Eine App konnte nicht zwischen den Applikationsformen unterscheiden und zeigte auch die größte Abweichung von der Umrechnung nach LONTS-Leitlinie. Die Konversion von transdermalem Fentanyl war in 13 von 17 Apps möglich. Dabei wurde im Median eine Zieldosierung von 20 mg (Minimum – Maximum 15–90 mg) berechnet. Eine Änderung der Darreichungsart von Hydromorphon i.v. in Hydromorphon p.o. war in 14 Apps möglich, es ergaben sich im Median 74 mg Hydromorphon (Minimum – Maximum 24–120 mg).

### Gefahrenhinweise der Apps

Vier Apps warnten den Benutzer vor einer möglichen Überdosierung bei hohen Eingangsdosierungen (Fall 16). Eine App wies nur bei der Umrechnung mit Methadon auf eine mögliche Fehldosierung aufgrund von variabler Pharmakokinetik hin. Zwei weitere Apps ermöglichten keine Umrechnung aus Methadon in Morphin mit Hinweis auf die variablen Umrechnungsfaktoren.

Auf die eventuell notwendige Reduktion der Zielopioiddosis unter Beachtung von inkompletter Kreuztoleranz wiesen 14 Apps hin, wovon 6 eine direkte prozentuale Reduktion ermöglichten.

Neun Apps wiesen des Weiteren darauf hin, dass die jeweilige App nicht Grundlage für eine ärztliche Behandlung sein darf und patientenindividuelle Unterschiede zur Dosierung eine entscheidende Rolle spielen. Zwei davon betonten explizit, dass der Opioidkonversion eine fachärztliche Konsultation zugrunde liegen sollte. Eine App unterschied bei keinem Opioid zwischen oraler, transdermaler oder intravenöser Applikationsform.

## Diskussion

In der vorliegenden Studie konnte gezeigt werden, dass Opioidumrechnungen mit vielen Apps möglich waren, sich jedoch eine eingeschränkte Anwendbarkeit und potenzielle Risiken zeigten. Des Weiteren wurden eine starke Streuung der Umrechnungsergebnisse und eine klinisch relevante Varianz der Dosierungen bei der Opioidumrechnung festgestellt [7]. Somit sind sowohl Über- als auch Unterdosierungen möglich. Dies deckt sich mit den Ergebnissen aus einer früheren Studie zu Opioidumrechnungs-Apps, die ebenfalls eine große Spannweite der Opioidumrechnungen in den verschiedenen getesteten Apps, insbesondere für Hydromorphon, feststellte [6]. Dies könnte unter anderem darin begründet sein, dass je nach Hersteller Angaben zur Morphinäquivalenz von 5:1 bis 10:1 vorliegen.

### Qualitative Bewertung von Apps zur Opioidumrechnung

Ein weiteres Problemfeld stellte die unklare Datengrundlage und Qualitätssicherung zahlreicher Apps dar. Keine der getesteten Apps wies eine Kennzeichnung als Medizinprodukt oder eine CE-Zertifikation als Qualitätssiegel auf. Dies entspricht früheren Erkenntnissen zum Stand der Qualitätssiegel von Apps im Gesundheitswesen [2] und bestätigt einen Bedarf bezüglich solcher Kennungen als Qualitätsindikatoren. Nur wenige Apps nannten eindeutige und nachvollziehbare Quellen für die Umrechnungsfaktoren. Des Weiteren wurden die Autoren der Apps oft nur unzureichend kenntlich gemacht, sodass Qualifikation, Kontext und Interessenkonflikte nicht transparent sind. Nur bei 10 Apps war das Mitwirken einer Ärztin bzw. eines Arztes erkennbar, obwohl dies die Güte der Umrechnungs-Apps verbessern könnte [6]. Somit scheint zumindest ein Großteil der Apps nicht den Standards für Opioidumrechnungen zu entsprechen, wie sie von Schnabel und Rittner vorgeschlagen wurden [15]. Darüber hinaus besteht ein Potenzial für Fehldosierungen durch verschiedene Applikationsformen, die beispielsweise bei einer App (pH-Medical Opioid Converter) nicht unterschieden wurden.

Nur wenige Apps nannten eindeutige und nachvollziehbare Quellen für die Umrechnungsfaktoren

Positiv zeigte sich hier jedoch, dass 9 Apps Gefahrenwarnungen beinhalteten oder eine fachärztliche Konsultation zur Opioidkonversion empfahlen (*N* = 2). Als ebenfalls praktisch erwiesen sich diejenigen Apps, die bei der Opioidrotation in Analogie zur Leitlinienempfehlung nach entsprechender Bestimmung der Äquivalenzdosis eine Reduktion der Zieldosierung vorschlugen [7, 14].

### Quellen der Opioidumrechnungen

Ein Teil der hohen Varianz der Opioidumrechnungen lässt sich durch die historische Entwicklung der Opioidumrechnungstabellen erklären. Diese Tabellen basieren teilweise auf klinischen Testungen mit mehrheitlich opioidnaiven Patient:innen, weshalb ein eventueller Toleranzeffekt mit Einfluss auf die relative analgetische Potenz nicht dargestellt wird [8]. Aus pharmakologischer Sicht problematisch bei der Umrechnung ist Methadon, das eine besondere Kinetik aufweist und dessen analgetische Potenz dosisabhängig ist [12]. Aufgrund der spezifischen Pharmakokinetik verfahren mehrere Apps besonders mit Methadon. Drei App-Autor:innen wiesen auf die Problematik hin, während eine App die Umrechnung ablehnt.

Die Problematik der eingeschränkten Quellenlage zeigte sich bereits in früheren Studien. In einer knapp zehn Jahre alten Studie zur Reliabilität von Smartphone-Apps für die Opioidkonversion konnte ebenfalls eine ausgeprägte Varianz der Umrechnungsfaktoren festgestellt werden [6]. Auch eine vom Bundesministerium für Gesundheit geförderte Studie wies bereits auf das Risikopotenzial nichtstandardisierter Apps im Gesundheitswesen hin [1]. Sinnvoll wäre es entsprechend der Empfehlung der AkdÄ, Äquivalenzdosierungen für Opioide in Beipackzetteln und Fachinformationen verfügbar zu machen [14]. Insbesondere neuere Wirkstoffe zeigten bereits in früheren Studien eine erhebliche Variabilität der oralen Morphinäquivalenz, wie es beispielsweise für Tapentadol diskutiert wurde [18]. Schlussendlich bleiben aufgrund des evidenzbasierten Konsensusprozesses Leitlinien ein geeignetes Werkzeug, Äquivalenzdosierungen für verschiedene Populationen zu definieren [7]. Beispielsweise könnte bei der Aktualisierung der S3-Leitlinie Akutschmerz auf besonders komplexe klinische Situationen eingegangen werden, wie die perioperative Akutschmerztherapie mit i.v.-Präparaten bei Patient:innen mit chronischer Opioidtherapie. Aus dieser Arbeit heraus könnten zudem von Fachgesellschaften evidenzbasierte Apps entwickelt werden, um schnell und sicher leitliniengerechte Entscheidungen zu ermöglichen [5, 11].

### Limitationen

Im Rahmen des Suchprozesses wurden Apps lediglich in deutscher und englischer Sprache eingeschlossen und die Suche zudem auf die ersten 50 Suchergebnisse bei Google® beschränkt. Es besteht dadurch die Möglichkeit, dass Opioidkonversions-Apps übersehen wurden. Weiterhin wurde durch den Ausschluss von herunterladbaren bzw. installationsbedürftigen Umrechnungsprogrammen für Computer nicht das gesamte Spektrum an digitalen Unterstützungsoptionen zur Opioidkonversion dargestellt.

Zudem erfolgte die Suche nach Smartphone-Apps über den iOS App Store® und den Google Play Store®. Diese stellen zwar aktuell die Marktführer für Apps dar [9], Umrechnungs-Apps anderer Plattformen können so jedoch übersehen werden. Des Weiteren ist limitierend zu nennen, dass der hier zugrunde gelegte Standard durch die aktuelle LONTS-Leitlinie definiert wurde und keine anderen Datenquellen Anwendung fanden [7]. Der Fokus dieser Leitlinie liegt auf der Langzeitopioidtherapie mittels oraler und transdermaler Applikationen, weshalb i.v.-Gaben und Behandlungen mit Substitutionspräparaten im Praxiswerkzeug nicht aufgeführt sind.

Weitere Aspekte, die bei der Opioidumrechnung berücksichtigt werden sollten, jedoch in den Apps eine unzureichende Entsprechung hatten und hier nicht abgebildet werden konnten, sind eine vorliegende relevante Organdysfunktion, demografische Faktoren sowie genetische Polymorphismen [10].

## Fazit für die Praxis

Durch die Prüfung verschiedener Testfälle für die Opioidkonversion aus der klinischen Praxis mithilfe digitaler Opioidkonversions-Apps konnten wir demonstrieren, dass die Dosierungen des Zielopioids variabel sind und damit eine potenzielle Gefährdung der Patientensicherheit nicht ausgeschlossen werden kann. Weiterhin konnte dargestellt werden, dass durch fehlende Qualitätsmerkmale und eingeschränkte Quellenlage eine adäquate Einschätzung der Apps erschwert wird. Insgesamt sind eine kritische Anwendung von Apps, eine Plausibilitätskontrolle und eine fachärztliche Konsultation zu empfehlen. Wünschenswert wäre die Mitentwicklung einer validierten App im Rahmen eines Leitlinienprozesses. Damit könnten Forderungen von digitalisierten Leitlinien umgesetzt und eine schnellere Informationsweitergabe ermöglicht werden.

## Supplementary Information




