# Supplementary material for: Support of opioid rotation using online apps: Evaluation of applicability and comparison to the LONTS guidelines
Source: Schmerz. 2022 Dec 12;37(4):290–6. [Article in German] doi: 10.1007/s00482-022-00683-5 (PMC10368547; doi:10.1007/s00482-022-00683-5)
Supplement: Supplementary file 1 [file 482_2022_683_MOESM1_ESM.pdf]

Online-Zusatzmaterial

Eingeschlossene Apps zur Umrechnung von Opioid-Analgetika

Nr.	Name	Autor:innen	Herkunft/Weblink
1	Opioid Calculator	ANZCA/Australian and New Zealand College of Anaesthetists, FPM/Faculty of Pain Medicine	iOS App Store®
2	Orthodose	Dr. V. Vandenhaute	iOS App Store®, Play Store
3	Opioid Rechner	Grünenthal GmbH	iOS App Store®
4	Palliative Care Tools	Nils Wommelsdorf	Google Play Store®
5	Opioid Converter	Vikas O'Reilly-Shah	Google Play Store®
6	Opioid Converter	Kirill Uryvaev	Google Play Store®
7	pH Medical Opioid Calculators	Philip Eagan	iOS App Store®
8	Opioid Dosage Conversion	Chris Marcellino MD	iOS App Store®
9	Opiatrechner	Universitätsspital Basel, Spital-Pharmazie	https://usb.x-service.ch/rechner/?sn=1&ts=1610709350691
10	Berechnung Äquivalenzdosen von Opiaten	Mircea Schneider	http://www.medicalapps.ch/EQD_Op_Ger.aspx
11	Pain Management	Chronic Pain Education Group	https://www.paindata.org/calculator.php

12	Opioid Calculator	Practical Pain Management; Remedy Health Media LLC	https://opioidcalculator.practicalpainmanagement.com/
13	Equivalent Opioid Calculator	CliniCalc LLC	https://clincalc.com/Opioids/
14	Opioid Conversion Calculator	Omni Calculator	https://www.omnicalculator.com/health/opioid
15	Opioid Equivalent Estimator	Pharmacy Joe / JosephMuench	https://www.pharmacyjoe.com/opioidequivalent-estimator/
16	Opioid Conversion Calculator Morphine Equivalents	Global RPH / David McAuley	https://globalrph.com/medcalcs/opioidpain-management-converter-advanced/
17	Opioid Conversion Calculator	eviQ -Cancer Institute NSW	https://www.eviq.org.au/clinical-resources/eviq-calculators/3201-opioid-conversion-calculator
